# Beyond Efficacy: Policy, Delivery, and Equity Determinants of Long-Acting Monoclonal Antibody Uptake for Infant RSV Prevention—A WAidid Consensus Document

**DOI:** 10.3390/vaccines14090739

**Published:** 2026-08-26

**Authors:** Susanna Esposito, Bahaa Abu-Raya, Brian Eley, Natasha Halasa, Federico Martinon-Torres, Asuncion Mejias, Vana Spoulou, Tobias Tenenbaum, Juan Pablo Torres, Albert Osterhaus, Octavio Ramilo, Nicola Principi

**Affiliations:** 1 Pediatric Clinic, Department of Medicine and Surgery, University Hospital of Parma, 43126 Parma, Italy; 2 Canadian Center for Vaccinology, IWK Health Centre and the Nova Scotia Health Authority, Dalhousie University, Halifax, NS B3K 6R8, Canada; bh723616@dal.ca; 3 Departments of Pediatrics, Dalhousie University, Halifax, NS B3K 6R8, Canada; 4 Departments of Microbiology and Immunology, Dalhousie University, Halifax, NS B3H 4R2, Canada; 5 Department of Paediatrics and Child Health, University of Cape Town, Cape Town 7530, South Africa; brian.eley@uct.ac.za; 6 Division of Pediatrics, Vanderbilt University Medical Center, Nashville, TN 37232, USA; natasha.halasa@vanderbilt.edu; 7 Translational Pediatrics and Infectious Diseases, Hospital Clínico Universitario de Santiago & Universidad de Santiago de Compostela, 15706 Santiago de Compostela, Spain; federico.martinon.torres@sergas.es; 8 Genetics, Vaccines and Infectious Diseases Research Group (GENVIP), Instituto de Investigación Sanitaria de Santiago de Compostela (IDIS), 15706 Santiago de Compostela, Spain; 9 Department of Infectious Diseases, St. Jude Children’s Research Hospital, Memphis, TN 38105, USA; asuncion.mejias@stjude.org (A.M.); octavio.ramilo@stjude.org (O.R.); 10 Immunobiology and Vaccinology Research Laboratory and Infectious Diseases Department “MAKKA”, First Department of Paediatrics, “Aghia Sophia” Children’s Hospital, Athens Medical School, 11527 Athens, Greece; vspoulou@med.uoa.gr; 11 Clinic for Child and Adolescent Medicine, Sana Klinikum Lichtenberg, Academic Teaching Hospital Charité-Universitätsmedizin Berlin, 10365 Berlin, Germany; tobias.tenenbaum@sana.de; 12 Instituto Sistemas Complejos de Ingeniería, Santiago 8370398, Chile; jptorres@uchile.cl; 13 Departamento de Pediatría y cirugía Pediátrica, Facultad de Medicina, Universidad de Chile, Santiago 8370398, Chile; 14 Research Center for Emerging Infections and Zoonoses, University of Veterinary Medicine Hannover, 30559 Hannover, Germany; albert.osterhaus@tiho-hannover.de; 15 Department of Pathophysiology and Transplantation, Università degli Studi di Milano, 20122 Milan, Italy; nicola.principi@unimi.it

**Keywords:** nirsevimab, respiratory syncytial virus, monoclonal antibodies, immunization coverage, implementation strategies, health equity

## Abstract

**Background:** Long-acting monoclonal antibodies have become an important strategy for preventing respiratory syncytial virus (RSV) disease in infants. Nirsevimab is the first product for which substantial post-licensure implementation data are available, whereas real-world evidence on clesrovimab remains limited. Although nirsevimab has demonstrated high efficacy, its uptake varies considerably across countries, healthcare systems, delivery settings, and population subgroups. This World Association for Infectious Diseases and Immunological Disorders (WAidid) consensus document examines the policy, organizational, economic, and equity-related determinants that shape real-world implementation of long-acting monoclonal antibodies for infant RSV prevention. **Methods:** This study was conducted as a structured narrative review and WAidid expert consensus document. A structured literature search was performed in PubMed and Embase for English-language publications relevant to nirsevimab uptake and implementation, complemented by targeted review of surveillance reports, policy documents, and public health guidance from the ECDC, UKHSA, and CDC, as well as reference lists of selected publications. Eligible sources included observational and real-world implementation studies, systematic reviews and meta-analyses, economic evaluations, guidelines, policy statements, surveillance reports, and relevant narrative reviews. Evidence was synthesized qualitatively according to policy frameworks, financing and reimbursement, delivery pathways, demographic and socioeconomic determinants, and healthcare-system factors influencing uptake. No statistical software was used because no quantitative re-analysis or meta-analysis was performed. **Results**: Nirsevimab uptake was strongly influenced by national RSV prevention policies, particularly whether countries adopted universal infant monoclonal antibody programs, maternal RSV vaccination strategies, dual maternal–infant approaches, or targeted risk-based models. Universal, publicly funded programs integrated into neonatal care achieved the highest and most homogeneous coverage, especially when administration occurred before hospital discharge and was supported by registry-based recall systems for infants born outside the RSV season. In contrast, fragmented, outpatient-only, insurance-dependent, or partially reimbursed models were associated with lower, delayed, or more variable uptake. Additional determinants included product cost, reimbursement pathways, provider practices, caregiver awareness and health literacy, insurance status, income, race and ethnicity, geographic deprivation, and access to primary pediatric care. Most available evidence comes from high-income countries, limiting generalizability to low- and middle-income settings, where RSV burden is greatest and implementation constraints may differ. **Conclusions:** Successful implementation of long-acting monoclonal antibodies for infant RSV prevention requires more than regulatory approval and demonstrated efficacy. Equitable uptake depends on clear national recommendations, sustainable public financing, reliable product supply, integration into neonatal and primary pediatric care, proactive identification and recall of eligible infants, and targeted strategies to reduce socioeconomic and geographic disparities. Although many determinants identified in high-income settings are likely relevant globally, their feasibility, relative importance, and impact require dedicated evaluation in low- and middle-income countries.

## 1. Introduction

Despite broad regulatory approval of nirsevimab and the more recent approval of clesrovimab as long-acting monoclonal antibodies for the prevention of respiratory syncytial virus (RSV) disease in infants [1,2,3], real-world implementation experience remains largely centered on nirsevimab. Its uptake has varied substantially across countries, regions, healthcare systems, and population subgroups, while coverage data for clesrovimab are still limited. The heterogeneity in uptake data of nirsevimab reflects differences in national implementation strategies, reimbursement models, delivery pathways, and access to care. A key determinant of this variability is the concurrent availability of maternal RSV immunization, which alters the population eligible for infant nirsevimab administration and may lead to partial substitution between preventive approaches, since most infants are expected to receive only one of the two interventions [4].

Importantly, implementation success should not be measured exclusively by crude coverage estimates. Timeliness of administration, reach among vulnerable populations, equity across socioeconomic groups, completeness of catch-up strategies, and the reduction of missed opportunities are equally important dimensions of successful implementation. Programs achieving similar overall coverage may therefore generate substantially different public health benefits depending on the quality and equity of delivery. Implementation fidelity, defined as the degree to which preventive interventions are delivered according to their intended design, has emerged as a major determinant of successful RSV prevention programs. High nominal coverage may coexist with suboptimal population protection when administration occurs late, when eligible infants are missed, or when important socioeconomic subgroups remain underrepresented.

Recent reviews and meta-analyses have shown that nirsevimab uptake is uneven even in settings where it is prioritized for infant RSV prevention, with coverage strongly shaped by national immunization policies, financing and reimbursement mechanisms, and whether programs are universal, publicly funded, or fragmented across regional, risk-based, private, or insurance-dependent pathways [5,6,7,8]. This review goes beyond the existing literature by integrating published evidence on these determinants with real-world implementation experience, emphasizing how policy design, funding structures, and delivery models interact to influence not only overall uptake but also timeliness, consistency, and equity of access.

This implementation challenge is particularly relevant to low- and middle-income countries (LMICs), where the burden of severe RSV disease is substantial but evidence on the real-world uptake and delivery of long-acting monoclonal antibodies remains limited. Health-system constraints in these settings—including product affordability, procurement and distribution capacity, shortages of trained healthcare personnel, variable access to antenatal and neonatal services, fragmented referral pathways, and limited surveillance and immunization registry infrastructure—may substantially influence both coverage and equity. Consequently, implementation models developed in high-income countries cannot be assumed to be directly transferable to LMICs. Identifying which determinants are context-specific and which represent broadly applicable implementation principles is therefore an important objective of this consensus document and a priority for future research.

The organization of delivery pathways is another major determinant of implementation success. Hospital-based administration at birth, particularly before discharge from maternity units, is generally associated with higher uptake than outpatient or multi-provider models, which require additional appointments, coordination, and availability of the product in the outpatient settings. Socioeconomic inequalities, geographic barriers, and disparities in access to healthcare further contribute to both between-country and within-country variability. In addition, differences in healthcare provider awareness, clinical practice patterns, communication with caregivers, and surveillance infrastructure may substantially influence program implementation and measured coverage across health systems.

Although the clinical efficacy and real-world effectiveness of nirsevimab have now been consistently demonstrated across randomized trials and multiple national implementation programs, substantial differences remain in the population-level impact achieved by different countries. These differences are no longer primarily explained by biological efficacy but rather by variation in implementation strategies, health-system organization, financing mechanisms, delivery pathways, and equitable access. Consequently, implementation has emerged as the principal determinant of the public health value of RSV passive immunization.

Understanding the drivers of disparities in nirsevimab uptake is essential to maximize the public health impact of this preventive intervention and to ensure equitable protection against RSV disease. Uneven coverage may limit reductions in RSV-related disease burden and hospitalizations, particularly among infants and communities already at increased risk of poor health outcomes. A structured characterization of these determinants can support targeted, context-specific interventions, guide resource allocation, and help translate the high efficacy demonstrated in clinical trials into real-world effectiveness. Accordingly, this World Association for Infectious Diseases and Immunological Disorders (WAidid) consensus document moves beyond describing international uptake estimates and aims to develop a comprehensive implementation framework for infant RSV prevention. We examine how policy decisions, healthcare delivery models, organizational capacity, provider behaviour, family-level determinants, and structural inequities interact to shape real-world uptake, timeliness, and ultimately the population impact of long-acting monoclonal antibodies. Finally, we identify knowledge gaps and propose priorities for future implementation research. The present consensus document specifically focuses on the acceptance, uptake, and implementation of long-acting monoclonal antibody prophylaxis for infant RSV prevention, with particular emphasis on nirsevimab because it currently accounts for the majority of available post-licensure implementation evidence. Maternal RSV vaccination is considered where relevant because national choices between maternal vaccination, infant monoclonal antibody prophylaxis, or dual strategies directly influence the population eligible for nirsevimab, its delivery pathways, and measured uptake. The objective of this document is therefore not to compare the effectiveness or coverage of all available RSV preventive strategies, but to identify the policy, organizational, economic, healthcare-system, and equity-related determinants of successful monoclonal antibody implementation. To provide a coherent multilevel interpretation of these determinants, this consensus document adopts a Policy–System–Provider–Recipient–Context framework, informed by established principles of implementation science. The framework recognizes that uptake is generated through interactions between upstream policy decisions, healthcare-system capacity, frontline provider behavior, caregiver and family-level factors, and the broader social and geographic context. Policy factors include eligibility, financing, procurement, and national preventive strategy; system factors include delivery pathways, registry infrastructure, supply, and care coordination; provider factors include knowledge, recommendation behavior, counseling, and responsibility for administration; recipient factors include awareness, acceptance, health literacy, socioeconomic position, and healthcare access; and contextual factors include geography, structural disadvantage, workforce availability, and healthcare infrastructure. These domains are interdependent rather than hierarchical, and weakness at one level may amplify barriers operating at another. The framework is used as an organizing structure for the narrative synthesis rather than as a formally validated scoring instrument.

## 2. Methods

### 2.1. Study Design and Scope

This manuscript was developed as a structured narrative review and expert consensus document of the WAidid. The purpose was not to conduct a systematic review or meta-analysis of the efficacy of nirsevimab, but rather to integrate the available evidence relevant to the real-world implementation of long-acting monoclonal antibodies for infant RSV prevention. Particular attention was given to factors influencing uptake, timeliness, delivery, financing, access, and equity.

Given the implementation-focused and narrative nature of the document, the review was not conducted according to a formal systematic-review protocol and was not intended to satisfy PRISMA reporting requirements. Consequently, a PRISMA flow diagram or other formal study-selection diagram was not generated.

### 2.2. Literature Search Strategy

A structured literature search was conducted in PubMed and Embase to identify publications relevant to nirsevimab and long-acting monoclonal antibody strategies for the prevention of RSV disease in infants. The search covered publications from 1 January 2015 through 31 March 2026. This starting date was selected to capture the period during which long-acting monoclonal antibodies for RSV prevention entered clinical development and to provide sufficient background preceding the regulatory approval and subsequent real-world implementation of nirsevimab while excluding older literature relating primarily to previous-generation monoclonal antibody prophylaxis that was outside the implementation-focused scope of this document. Only English-language sources were considered.

The search combined free-text terms and controlled vocabulary, including Medical Subject Headings (MeSH) where applicable, related to RSV, nirsevimab, long-acting monoclonal antibodies, infants, implementation, uptake, coverage, effectiveness, reimbursement, access, policy, and health equity. The principal search concepts were combined using Boolean operators as follows: (“respiratory syncytial virus” OR RSV) AND (nirsevimab OR “long-acting monoclonal antibody” OR “monoclonal antibody”) AND (infant OR newborn OR neonate) AND (uptake OR coverage OR implementation OR effectiveness OR access OR equity OR reimbursement OR policy).

Search syntax was adapted as necessary to the indexing structure and controlled vocabulary of each database. Reference lists of relevant reviews, guidelines, policy documents, and eligible primary studies were also examined to identify additional pertinent sources.

### 2.3. Retrieval of Grey Literature and Official Guidance

Because several aspects of nirsevimab implementation, including national recommendations, coverage estimates, reimbursement arrangements, program organization, and surveillance findings, may be reported first or exclusively by public health authorities, the bibliographic database search was complemented by a targeted search of official public health sources.

The websites and document repositories of the European Centre for Disease Prevention and Control (ECDC), UK Health Security Agency (UKHSA), and US Centers for Disease Control and Prevention (CDC) were searched for English-language documents available up to 31 March 2026. Searches used combinations of the terms “RSV”, “respiratory syncytial virus”, “nirsevimab”, “monoclonal antibody”, “infant”, “immunization”, “immunisation”, “coverage”, “uptake”, “recommendation”, “guidance”, “surveillance”, “implementation”, and “policy”. Relevant agency webpages, surveillance reports, recommendations, policy documents, and implementation guidance were reviewed. References and links contained within relevant official documents were also followed when they provided additional information directly related to the objectives of this review.

Only documents issued or hosted by the relevant official agencies and containing substantive information on RSV prevention policy, implementation, uptake, coverage, delivery, reimbursement, or access were retained.

### 2.4. Eligibility Criteria and Source Selection

Eligible sources included phase III randomized or controlled clinical trials when relevant to the clinical context of implementation; observational and multicenter studies; real-world effectiveness and implementation studies; population-based and surveillance studies; systematic reviews and meta-analyses; economic evaluations; practice guidelines; policy statements; public health surveillance reports; and relevant narrative reviews.

Phase I and phase II studies were not considered eligible because their primary objectives are generally pharmacokinetic, safety, dose-finding, or immunogenicity assessment under controlled conditions and they do not directly address the implementation outcomes that were the focus of this document, including real-world uptake, delivery pathways, reimbursement, access, and equity.

Sources were considered relevant when they provided information on one or more of the following domains: nirsevimab use or uptake; immunization coverage and timeliness; real-world implementation or effectiveness; national or regional RSV prevention policies; financing and reimbursement; procurement and product availability; hospital, outpatient, pharmacy, or primary-care delivery pathways; caregiver or healthcare-provider determinants; demographic and socioeconomic determinants of uptake; geographic disparities; or other factors influencing equitable access.

Publications were excluded when they were not written in English; did not concern RSV prevention in infants or young children; focused exclusively on interventions unrelated to monoclonal antibody prophylaxis or maternal RSV immunization; did not provide information relevant to implementation, uptake, coverage, access, policy, reimbursement, or equity; or contained neither original data, substantive policy or surveillance information, nor a synthesis relevant to the objectives of the review.

Clesrovimab was not a primary focus of the synthesis because its introduction was more recent and real-world evidence regarding uptake, delivery, coverage, reimbursement, and equity remained limited within the search period. Accordingly, the implementation analysis centered primarily on nirsevimab, for which substantially more post-licensure evidence was available.

A total of 50 sources meeting these relevance criteria were retained for the qualitative narrative synthesis.

### 2.5. Evidence Extraction, Appraisal, and Narrative Synthesis

Relevant information was extracted from the selected sources according to the principal domains of the review: national policy frameworks; financing and reimbursement; procurement and supply; organization of delivery; timing of administration; demographic determinants; socioeconomic determinants; caregiver and provider factors; healthcare-system characteristics; coverage; and equity of access.

For analytical purposes, extracted determinants were additionally organized according to five interacting levels: Policy, System, Provider, Recipient, and Context. This structure was used to integrate heterogeneous evidence across policy documents, real-world studies, observational cohorts, economic evaluations, and implementation reports. It was informed by multilevel implementation science frameworks, including CFIR and RE-AIM, but these frameworks were not formally applied because study selection and data extraction were not prospectively designed around their individual constructs. The purpose of this framework was therefore organizational and interpretive rather than quantitative.

The evidence informing the different implementation domains was heterogeneous and was therefore interpreted according to its directness to the outcome of interest. Population-based surveillance, large observational cohorts, and real-world implementation studies were considered the most direct sources for observed uptake, timeliness, and disparities. Smaller observational studies were used principally to identify individual-, provider-, or local system-level determinants. Economic evaluations and modeling studies were interpreted as evidence regarding affordability, budget impact, and potential sustainability rather than as direct evidence of achieved coverage. National guidelines, policy statements, and public health documents were used to characterize eligibility rules, financing arrangements, delivery models, and program organization. Accordingly, findings from these different source categories were integrated qualitatively but were not treated as equivalent levels of evidence.

Because the included evidence encompassed markedly different source types—including randomized studies, observational studies, surveillance data, implementation studies, economic evaluations, systematic reviews, public health guidance, and policy documents—a single formal risk-of-bias instrument was not considered applicable across all sources. Accordingly, ROBINS-I, the Newcastle–Ottawa Scale, and GRADE were not formally applied. Instead, evidence was appraised narratively, taking into consideration study design, population size and representativeness, source and completeness of outcome data, relevance to real-world implementation, consistency with findings from other settings, and directness to the implementation questions addressed in this document. Findings derived from individual or geographically restricted studies were interpreted more cautiously than findings supported by national surveillance systems, large population-based studies, or consistent observations across multiple settings.

No quantitative pooling was undertaken. A formal meta-analysis was considered inappropriate because of substantial heterogeneity among the available sources in study design, populations, countries and healthcare systems, eligibility criteria, implementation strategies, delivery models, definitions of coverage and uptake, follow-up periods, and outcome measures. The evidence was therefore synthesized qualitatively to identify recurrent determinants of implementation success, barriers to uptake, sources of inequity, and potentially transferable organizational and policy approaches.

No dedicated statistical software was used because no quantitative re-analysis or meta-analysis was performed; evidence extraction, categorization, and narrative synthesis were conducted manually.

### 2.6. Development of the WAidid Consensus

The evidence identified through the structured narrative review provided the basis for the development of the WAidid consensus statements and implementation framework. The consensus process was designed to integrate the published evidence with the multidisciplinary expertise and international implementation experience of the participating authors.

An initial draft identifying the principal policy, organizational, economic, demographic, and equity-related determinants of nirsevimab implementation was prepared on the basis of the literature synthesis and circulated among all members of the WAidid expert group. The document and its proposed conclusions were subsequently reviewed through iterative rounds of written comments and revisions. Areas of disagreement or uncertainty were discussed among the authors, and the wording was modified until agreement was reached. No formal Delphi procedure or anonymous numerical voting system was used. Consensus was defined as agreement of all participating authors with the final statements and recommendations included in the manuscript. Recommendations were formulated when they were supported by consistent evidence from the literature and/or convergent implementation experience across healthcare settings; areas for which the available evidence was limited or context-dependent were presented as knowledge gaps or priorities for future research rather than as definitive recommendations.

All authors reviewed and approved the final version of the consensus document.

## 3. Implementation of Nirsevimab Use: The Role of National Policies

The geographical distribution of the available implementation evidence was markedly uneven. Most published post-licensure data on nirsevimab uptake, delivery, reimbursement, and equity originated from the United States and Western European countries, with additional population-level evidence from Chile. Comparable real-world data from Africa, Asia, and many countries in Latin America were scarce or unavailable during the review period. The country examples presented below should therefore be interpreted as evidence from settings with early or relatively mature implementation programs rather than as globally representative models. Their applicability to other health systems, particularly LMICs, requires context-specific evaluation.

National policy is arguably the strongest upstream determinant of successful RSV prevention programs [5,6,9,10,11,12,13,14,15,16,17,18,19,20,21,22,23,24,25,26,27,28]. Policy decisions define not only who should receive immunoprophylaxis but also how products are financed, procured, distributed, delivered, monitored, and ultimately accepted by healthcare providers and families. Consequently, apparently similar biological interventions may generate markedly different public health impacts depending on the policy framework under which they are implemented. Policy frameworks differ across several dimensions that extend beyond the simple choice between maternal vaccination and infant monoclonal antibodies [5,6,9,10,11,12,13,14,15,16,17,18,19,20,21,22,23,24,25,26,27,28]. These include target population definition, reimbursement mechanisms, procurement models, delivery platforms, governance structure, implementation timelines, and monitoring systems. The combination of these components constitutes the implementation architecture of RSV prevention programs and largely determines their capacity to achieve high, timely, and equitable coverage [5,6,9,10,11,12,13,14,15,16,17,18,19,20,21,22,23,24,25,26,27,28]. In settings where maternal RSV immunization has been prioritized, or where decisions regarding RSV prevention have been left largely to individual providers and caregivers, nirsevimab use has generally remained limited or marginal [6,9,10,11,12].

In the UK, policy decisions adopted in 2023 favored the implementation of maternal RSV immunization over the universal use of long-acting monoclonal antibodies in infants. This approach was supported by considerations related to expected cost, integration within existing antenatal immunization platforms, and the potential to provide protection from birth without dependence on postnatal healthcare access [9]. In particular, the Joint Committee on Vaccination and Immunisation (JCVI) recommended the development of a national RSV prevention program prioritizing vaccination during pregnancy rather than universal neonatal administration of nirsevimab [10]. Consequently, nirsevimab was not implemented as a population-level intervention during the 2023–2024 RSV season, and its uptake in the UK remained negligible. Consistent with this, a large systematic review and meta-analysis of nirsevimab uptake, including more than 1.3 million individuals across multiple countries, did not report UK population-level uptake data, reflecting the absence of a universal infant program in this setting [6]. By contrast, uptake of maternal RSV vaccination increased progressively, exceeding 50% among pregnant women delivering in mid-2025, with coverage of 55.6% in July 2025. However, substantial regional and sociodemographic heterogeneity persisted, with coverage ranging from 64.4% in the Southeast to 47.3% in London, and from 78.5% among women of Chinese ethnicity to 30.5% among Black or Black British Caribbean women [11]. The first documented introduction of nirsevimab within the National Health Service occurred only in the 2025–2026 RSV season and was limited to a targeted, risk-based program for a restricted population of high-risk infants, including those born before 32 weeks’ gestation, estimated at approximately 7000–9000 infants annually [12].

In the USA, RSV prevention policy for infants has been structured as a dual-strategy model, integrating maternal immunization and nirsevimab. This approach is supported by national recommendations from the Centers for Disease Control and Prevention (CDC) [13] and the American Academy of Pediatrics (AAP) [14]. These recommendations specify that either maternal vaccination during pregnancy or nirsevimab administration to the infant should be used to prevent severe RSV disease, with most infants expected to receive only one of the two interventions rather than both. For infants born to vaccinated mothers, nirsevimab is recommended when birth occurs within 14 days of maternal immunization, a period during which transplacental antibody transfer may be incomplete. In addition, a single dose of nirsevimab is recommended for children entering their second RSV season who remain at increased risk of severe disease, thereby extending protection to selected high-risk populations beyond the first year of life.

The US experience demonstrates that combining maternal immunization with infant monoclonal antibodies substantially increases implementation complexity. Healthcare providers must evaluate maternal vaccination status, timing of vaccination, gestational age, infant age, eligibility criteria, insurance coverage, and product availability before recommending immunization. Each additional decision node increases the probability of delayed administration or missed opportunities. Initial implementation in the United States also highlighted the critical importance of supply chain resilience. Temporary shortages affected provider confidence, delayed administration, complicated inventory management, and generated regional inequities. These observations emphasize that manufacturing capacity and procurement planning represent integral components of implementation success rather than external logistical issues. During the first year of implementation in the USA, uptake remained suboptimal and highly heterogeneous, reflecting variability in program delivery, supply, financing, and access across states and healthcare systems. National surveillance during the 2023–2024 RSV season showed that only 29% of infants were protected through either maternal vaccination or nirsevimab, with approximately 18.5–19% receiving nirsevimab directly [15,16]. Geographic variability was substantial: overall RSV prevention coverage ranged from approximately 11% in lower-performing jurisdictions to more than 50% in higher-performing states, while nirsevimab-specific uptake varied between 6.5% and 34.9% across regions [16]. Differences were also observed in the timing of administration, with only about 38% of infants receiving nirsevimab within the first week of life, partly because of early supply constraints. Temporal variation within the same RSV season further highlighted the dynamic interaction between maternal vaccination and infant monoclonal antibody use. In a large integrated healthcare system, approximately 75% of infants born at the beginning of the season (October 2023) received nirsevimab compared with less than 30% of those born later, between January and March 2024, coinciding with increasing uptake of maternal vaccination [17]. In subsequent seasons, however, coverage improved substantially. During the 2024–2025 RSV season, approximately 47.5% of eligible infants received nirsevimab, while overall RSV immunization coverage, combining maternal vaccination and monoclonal antibody use, reached approximately 66% [5]. Early surveillance data from the 2025–2026 season suggest continued expansion of coverage, although comprehensive population-level estimates specifically for nirsevimab remain limited [18].

Across Europe, national policies have increasingly favored universal infant immunization with long-acting monoclonal antibodies. Despite differences in healthcare organization, financing mechanisms, and population size, successful European programs shared several common characteristics. These included universal publicly funded eligibility, centralized procurement, integration into neonatal care pathways, administration before hospital discharge whenever possible, and structured catch-up mechanisms for infants born outside the RSV season. During the 2025–2026 season, most countries, including Austria, Belgium, Cyprus, Czechia, Finland, France, Germany, Greece, Iceland, Ireland, Liechtenstein, Luxembourg, the Netherlands, Portugal, Spain, and Sweden, adopted universally funded and fully reimbursed infant immunization programs for RSV prevention [19]. These programs are generally delivered seasonally and target all infants below a defined age threshold, commonly younger than 6–8 months at the beginning of or during the RSV season, irrespective of underlying risk conditions. Additional provision is usually made for selected high-risk children entering their second RSV season, including those with extreme prematurity, chronic lung disease, hemodynamically significant congenital heart disease, or severe immunocompromise. In only a few countries, such as Denmark, Latvia, and Lithuania, monoclonal antibody use was restricted to infants at increased risk of severe RSV disease [19].

Several countries with funded infant programs also introduced catch-up strategies, mainly to protect infants entering their first RSV season who had been born outside the seasonal immunization campaign. These included Belgium, Cyprus, Czechia, Finland, Germany, Iceland, Ireland, Latvia, Liechtenstein, Luxembourg, the Netherlands, Spain and Portugal [19]. In contrast, funded maternal RSV vaccination programs have been implemented less consistently across Europe in the post-licensure phase. Five EU/EEA countries (Belgium, Cyprus, France, Greece, and Luxembourg) have introduced both infant monoclonal antibody programs and maternal vaccination, allowing pregnant individuals to choose between vaccination during pregnancy and postnatal administration of monoclonal antibody to their newborn. Conversely, only three countries, Romania, Poland, and Slovenia, have implemented government-funded maternal RSV vaccination programs exclusively [19].

In several European countries, universal or near-universal policies have been associated with rapid achievement of high coverage. Spain represents the most comprehensive and consistently reported European experience. Universal immunization strategies introduced during the 2023–2024 RSV season achieved very high coverage, with national estimates approaching 90% of eligible infants and multicenter surveillance showing regional coverage ranging from 79% to 99% [20,21]. Population-based data from the NIRSE-GAL study in Galicia documented that 11,796 of 12,492 eligible infants received nirsevimab, corresponding to 94.4% coverage, with sustained high uptake during follow-up [22]. The Galician experience further illustrates the value of integrating implementation with digital health infrastructure. Centralized appointment systems, real-time monitoring of eligible infants, proactive recall of out-of-season births, and continuous surveillance of effectiveness and safety created a learning healthcare system capable of continuously optimizing programme performance [23]. Similar results were reported in Murcia, where 11,785 of 12,606 infants were immunized during the 2024–2025 campaign, corresponding to 93.5% overall coverage. Uptake was particularly high among infants immunized at birth, reaching 96.0%, compared with 90.5% among those receiving delayed administration [24]. Spain provides one of the clearest demonstrations that implementation strategy can be as important as biological efficacy. The combination of universal public funding, centralized procurement, maternity-based administration, high professional acceptance, and coordinated regional implementation generated consistently high coverage across multiple autonomous communities despite organizational differences. This suggests that successful implementation depends less on individual healthcare settings than on coherent system-wide organization.

Early Italian experiences illustrate the transition from initial regional implementation to more mature large-scale programs. In Valle d’Aosta, the first Italian region to implement universal nirsevimab prophylaxis during the 2023–2024 season, approximately 69–71% of eligible neonates received nirsevimab, corresponding to 369 of 556 infants, suggesting moderate uptake during the initial rollout [25]. However, subsequent expansion to more structured regional programs yielded substantially higher coverage. In Tuscany, universal immunization with nirsevimab introduced during the 2024–2025 season achieved approximately 90–90.5% coverage across both in-season and out-of-season birth cohorts [26].

By contrast, other European countries have reported more moderate or heterogeneous uptake. France may be considered a moderate-uptake setting, characterized by early national adoption of nirsevimab but incomplete coverage during the first implementation season. In a national ambulatory test-negative case–control study conducted during the 2023–2024 RSV season, the proportion of infants who had received nirsevimab was substantially higher among RSV-negative controls than among RSV-positive cases, 41.2% versus 13.7%, respectively. This finding highlights the coexistence of moderate population uptake with high individual-level effectiveness during the early phase of implementation [27]. In Luxembourg, national estimates suggested high neonatal coverage of approximately 84%; however, case-based analyses revealed substantial heterogeneity across maternity wards, with coverage ranging from 66% to 94% [28].

Overall, these examples indicate that national policy decisions are a major determinant of nirsevimab uptake. Universal, publicly funded programs integrated into existing maternal, neonatal, or pediatric care pathways are more likely to achieve rapid and homogeneous coverage. In contrast, risk-based strategies, fragmented delivery models, partial reimbursement, or competing preventive options without clearly defined implementation pathways may result in lower or more uneven uptake, even when nirsevimab is licensed and clinically recommended.

The experience of different countries shows that nirsevimab uptake is closely linked to the policy framework adopted for RSV prevention. Universal infant programs, dual maternal–infant strategies, risk-based approaches, and regionally implemented programs differ substantially in their operational requirements and expected coverage. The main policy models and their implications for nirsevimab uptake are summarized in Table 1.

## 4. External Factors Influencing Immunization Coverage

Data from countries in which maternal RSV immunization is the predominant preventive strategy, and in which monoclonal antibodies are generally reserved for infants at highest risk or for those not protected by maternal vaccination, clearly indicate that nirsevimab uptake is strongly shaped by national RSV prevention policies. However, even in countries where monoclonal antibodies represent the preferred strategy for preventing RSV disease in infants, uptake is influenced by multiple additional factors that may substantially affect overall coverage, timely administration, and equity of access. The most relevant of these determinants are discussed below.

### 4.1. Organizational Factors

Multiple large-scale implementation programs provide convergent evidence that the highest nirsevimab coverage is achieved when administration is embedded within routine neonatal care pathways, particularly through in-hospital delivery shortly after birth. This model has clear operational advantages over outpatient or fragmented approaches, as it reduces missed opportunities. These observations are consistent with broader experience from established immunization programs, in which coverage depends not only on acceptance of the preventive product but also on the number and complexity of contacts required to receive it. Birth-dose programs, particularly hepatitis B and Bacillus Calmette–Guérin vaccination, provide the closest operational analogy to nirsevimab administration during the birth hospitalization: integration into routine neonatal care reduces dependence on caregiver-initiated follow-up and limits missed opportunities. Other programs, including rotavirus and HPV vaccination, likewise illustrate the broader principle that convenient access, clear provider responsibility, reliable supply, reminder systems, and reduction of financial and logistical barriers can influence uptake. However, because these vaccines target different ages and use different delivery platforms and schedules, they are considered supportive implementation analogies rather than directly comparable models for nirsevimab delivery. A particularly illustrative example is Chile’s national immunization program, NIRSE-CL, the first universal rollout in the southern hemisphere [29]. This program achieved coverage exceeding 94%, largely attributable to the integration of nirsevimab into routine neonatal care and the national immunization infrastructure. Importantly, administration occurred very early after birth, with a median time to immunization of approximately 1 day in the seasonal birth cohort.

Supportive evidence also comes from France, where early implementation studies conducted in maternity units showed that in-hospital administration was feasible and well accepted, with uptake exceeding 90% among eligible newborns [30]. National program data further indicate that nirsevimab was frequently administered before discharge from maternity wards, reinforcing the effectiveness of hospital-based delivery models in ensuring early protection and minimizing missed opportunities [30].

Hospital-based administration improves implementation through multiple independent mechanisms rather than through a single organizational advantage [31,32]. First, it minimizes loss to follow-up by reaching infants before discharge. Second, it eliminates the need for caregiver-initiated healthcare seeking. Third, it reduces scheduling complexity and transportation barriers. Fourth, it standardizes eligibility assessment under neonatal care teams. Finally, it allows centralized stock management and immediate documentation within electronic medical records. These mechanisms operate synergistically to improve both coverage and timeliness.

Programs relying primarily on outpatient administration or fragmented delivery pathways tend to show lower or more variable uptake. Every additional day between birth and immunization represents a potential period of vulnerability during which infants remain susceptible to RSV infection. Consequently, implementation strategies should aim not only to maximize overall coverage but also to minimize the interval between birth and passive immunization. Immunization of infants not following discharge from the hospital requires several additional steps, including scheduling outpatient visits, ensuring parental adherence to appointments, coordinating across multiple levels of care, and maintaining consistent product availability. Each of these steps may introduce delays or missed opportunities, thereby reducing both timely protection against t RSV and overall coverage [31,32].

Nevertheless, a structured combination of in-hospital neonatal administration and organized follow-up for catch-up cohorts can maximize coverage and population benefit. A well-documented example is the NIRSE-GAL program in Galicia, Spain [22]. In this program, infants born during the RSV season were immunized in hospital on the first day of life, whereas infants born before the season were systematically identified through population registries and invited for scheduled administration in hospitals or healthcare centers. This coordinated model resulted in very high overall coverage, approximately 94.4%, and was associated with substantial reductions in RSV-related hospitalizations during the first season. Data from Tuscany in Italy also support the value of a combined strategy, showing that coordinated prophylaxis was associated with the greatest reduction in RSV-related hospitalizations [33].

Beyond national recommendations, the way nirsevimab is delivered in practice is critical to program success. Current evidence shows that timely and equitable coverage is best supported by integration into routine neonatal care, administration before hospital discharge, registry-based recall systems, and close coordination with primary pediatric services. The main organizational determinants of nirsevimab implementation are summarized in Table 2.

The effectiveness of combined delivery models depends on strong coordination across all stages of implementation. If registries are not integrated, referral pathways are unclear, communication between maternity units and primary care providers is delayed, or product distribution is unreliable, outpatient and community-based delivery may become inconsistent, leading to delays, missed opportunities, and variable coverage. France provides an example of this challenge: nirsevimab distribution was extended beyond hospitals to pharmacies, creating multiple access points. Although this approach may improve availability, community-based pathways require prescribing, dispensing, and administration, which may generate regional and provider-level variability in uptake [34].

### 4.2. Demographic Factors

Infant characteristics and timing of birth relative to the RSV season significantly influence access to nirsevimab. In a large USA pediatric primary care network including 7208 eligible infants, age was a strong determinant of uptake: each additional month of age from birth was associated with a markedly lower likelihood of receiving nirsevimab (odds ratio [OR], 0.60 per month; 95% confidence interval [CI], 0.58–0.62) [7]. This finding suggests that the earliest opportunities for administration are critical and that delays after birth may rapidly translate into missed prophylaxis.

Perinatal and biological characteristics also influence uptake. Data from the French national immunization campaign, which included approximately 328,000 eligible infants, showed that prioritization of clinically vulnerable groups was reflected in real-world uptake patterns [35]. Very preterm infants (born before 32 weeks of gestation) had significantly higher odds of receiving nirsevimab than term infants (adjusted OR [aOR], 2.07; 95% CI, 1.82–2.37). Male sex was also independently associated with receipt (aOR, 1.07; 95% CI, 1.05–1.10), as was season of birth. Infants born in June–July were more likely to be immunized than those born earlier in the year, such as February–March (aOR, 1.69), reflecting the interaction between clinical prioritization, campaign timing, and proximity to the RSV season.

Race and ethnicity have also emerged as major determinants of disparity in real-world implementation. In the study by Ahmed et al. [7], black infants had significantly lower odds of receiving nirsevimab than white infants (OR, 0.53; 95% CI, 0.43–0.65), even after adjustment for age, insurance status, and neighborhood characteristics. These disparities occurred despite near-universal product availability, underscoring the role of structural and healthcare-system factors in producing inequitable access.

### 4.3. Economic Factors

Economic constraints remain a central barrier to the implementation of universal nirsevimab programs, although different economic components contribute unequally to limiting uptake and scalability. The most important factor is acquisition cost and overall budget impact, which directly influence whether countries can extend prophylaxis to an entire birth cohort. Economic evaluations consistently indicate that universal strategies become sustainable only below defined price thresholds. In Italy, modelling studies estimated that nirsevimab would meet commonly accepted willingness-to-pay thresholds only at a price of approximately €267–€400 per dose, with higher prices rapidly reducing cost-effectiveness and limiting feasibility at the population level [36]. Similar analyses confirm that cost-effectiveness is highly sensitive to acquisition price, assumptions regarding RSV disease burden, and baseline risk profiles, with less favorable results when prophylaxis is extended to low-risk infants [9,37]. In the USA, this constraint is particularly evident: universal immunization has been associated with an incremental cost-effectiveness ratio of approximately $153,517 per quality-adjusted life-year gained, exceeding conventional willingness-to-pay benchmarks used in many healthcare systems and thereby favoring selective or seasonal strategies over universal adoption [38]. More recent evaluations in the US similarly show that cost-effectiveness estimates are highly dependent on drug pricing, disease burden assumptions, and healthcare utilization patterns [39].

Beyond acquisition cost, health system-level delivery and reimbursement mechanisms represent major determinants of coverage and equity. The economic burden of implementation is not limited to procurement but also includes distribution, storage, inventory management, staff time, and administrative complexity, all of which may disproportionately affect less-resourced healthcare settings. Reimbursement structures are particularly important. In a USA population-based modelling study, a pharmaceutical-type reimbursement pathway, characterized by insurance-dependent coverage, prior authorization requirements, and restrictive eligibility criteria, resulted in substantially lower population coverage than a vaccine-like delivery model [40]. Transitioning to a programmatic, vaccine-like system increased coverage by 32% overall and disproportionately benefited vulnerable groups, including a 44% greater reduction in hospitalizations and emergency visits among publicly insured infants. These findings demonstrate how reimbursement and delivery frameworks can either amplify or reduce health inequities. This issue is especially relevant in private practice settings, where financial risk, delayed reimbursement, and uncertainty regarding payment may discourage providers from stocking and administering nirsevimab [41].

Socioeconomic heterogeneity in access, particularly related to insurance and income, represents a further barrier even when the product is available for a nominal price. Direct economic indicators remain strong predictors of uptake. In a large USA cohort, infants with public insurance had lower adjusted odds of receiving nirsevimab than those with private insurance (aOR, 0.80; 95% CI, 0.70–0.89) [5]. Similarly, survey-based data showed that higher household income, defined as ≥$100,000, and private insurance coverage were associated with significantly higher intention to receive nirsevimab (OR, 2.59; 95% CI, 1.07–6.22 and OR, 3.03; 95% CI, 1.27–7.23, respectively) [8]. Consistent results were reported in a large primary care network including 7208 eligible children younger than 8 months, where only 35% of eligible infants received nirsevimab despite near-universal availability, and public insurance remained an independent negative predictor of receipt (OR, 0.79; 95% CI, 0.67–0.92) [7]. These findings reinforce the role of financial factors in shaping access.

### 4.4. Indirect Socioeconomic Factors

Disparities in nirsevimab uptake are not solely attributable to direct economic barriers. These also reflect indirect socioeconomic determinants, including health literacy, caregiver engagement with healthcare systems, perceived risk of infection, and community-level deprivation. These factors interact and may persist even in settings where nirsevimab is broadly available, highlighting the multifactorial nature of inequitable access.

Household exposure to healthcare environments appears to facilitate uptake [8]. Having a parent or partner employed in healthcare, which may serve as a proxy for health literacy and familiarity with preventive interventions, was among the strongest predictors of intention to receive nirsevimab. Intended uptake was reported by 74.2% of caregivers with a healthcare-affiliated household member, compared with 57.7% of those without such exposure (OR, 4.29; 95% CI, 1.75–10.5; *p* = 0.001). Findings from a French single-center study further support the importance of prior engagement with healthcare providers [42]. In a cohort of 1361 infants evaluated during the first national nirsevimab immunization campaign, adherence to at least one recommended vaccine during pregnancy was independently associated with acceptance of passive immunization in newborns. Conversely, socioeconomic vulnerability was associated with lower caregiver intention to accept RSV immunoprophylaxis. In particular, participation in the Women, Infants, and Children program, used as a marker of economic and social disadvantage, was linked to a significantly lower likelihood of intending to receive nirsevimab (OR, 0.35; 95% CI, 0.13–0.91; *p* = 0.03). This suggests that barriers such as limited access to information, healthcare navigation challenges, and broader structural disadvantage may influence caregiver decision-making.

Behavioral and exposure-related factors further modulate the uptake of nirsevimab. In the study by Lipsett et al. [8], children attending childcare had higher odds of intended nirsevimab receipt (OR, 2.85; 95% CI, 0.94–8.65; *p* = 0.06) and significantly higher odds of actual uptake (OR, 3.49; 95% CI, 1.35–8.98; *p* = 0.01). In the same cohort, overall uptake was 66.9%, and 74.5% of caregivers expressed support for nirsevimab immunoprophylaxis for their children by reporting an intention to receive it. Childcare attendance emerged as a key behavioral driver of translation from intention to action, whereas no other factor was significantly associated with actual uptake. These findings suggest that families perceiving a higher risk of RSV exposure, or those with more structured contact with healthcare and childcare systems, may be more likely to access novel preventive interventions.

Community-level deprivation further amplifies inequities. In the study by Ahmed et al. [7], receipt of nirsevimab was independently associated with neighborhood opportunity. Infants residing in areas with a very low Child Opportunity Index, a composite measure incorporating income, educational attainment, environmental conditions, and social resources, had significantly lower odds of receiving nirsevimab than those living in high-opportunity areas (OR, 0.70; 95% CI, 0.54–0.91), even after adjustment for race, insurance status, and age. Complementary geospatial analyses using the Area Deprivation Index support this gradient. In a USA cohort of 667 infants admitted to the neonatal intensive care unit, neighborhood deprivation was associated with differences in the timing and likelihood of RSV immunoprophylaxis. Infants from lower-deprivation neighborhoods were more likely to have received prophylaxis before hospitalization, whereas those from higher-deprivation areas were more likely to receive it only after admission or to remain unprotected, suggesting delayed or reduced access among more socioeconomically disadvantaged groups [16].

Together, these findings indicate that early uptake of nirsevimab tends to cluster in more advantaged families and communities. This pattern reflects structural inequities in healthcare access, health literacy, care coordination, and preventive service delivery. Addressing these disparities requires not only adequate funding and product availability, but also integrated delivery systems, proactive outreach, reliable recall mechanisms, and targeted communication strategies for populations at greatest risk of being missed.

Taken together, the evidence indicates that suboptimal nirsevimab uptake is rarely attributable to a single barrier. Instead, it results from the interaction of policy, financing, delivery pathways, healthcare access, socioeconomic position, caregiver awareness, and surveillance capacity. These determinants, their mechanisms, and potential corrective strategies are summarized in Table 3.

### 4.5. Integrated Conceptual Synthesis of Determinants of Uptake

Taken together, the available evidence supports a multilevel interpretation of nirsevimab uptake in which determinants operate interactively rather than independently. At the most upstream level, national policy establishes eligibility, financing, procurement, and the relationship between maternal vaccination and infant monoclonal antibody prophylaxis [5,6,9,10,11,12,13,14,15,16,17,18,19,20,21,22,23,24,25,26,27,28]. These decisions define the implementation environment within which healthcare organizations operate and can substantially influence the size of the eligible population, the delivery setting, and the overall level of nirsevimab uptake.

At the health-system and organizational level, procurement reliability, reimbursement, product availability, integration into maternity and neonatal care, registry infrastructure, catch-up mechanisms, and coordination with primary pediatric services determine whether eligible infants can be reached efficiently and at the appropriate time [9,22,29,30,31,32,33,34,36,37,38,39,40,41]. Programs integrating administration into routine neonatal care and providing nirsevimab before hospital discharge have consistently achieved high and timely coverage, whereas fragmented or multistep outpatient pathways may increase the risk of delays and missed opportunities [22,29,30,31,32,33,34].

At the individual and family level, demographic characteristics, timing of birth relative to the RSV season, socioeconomic position, insurance status, geographic access, caregiver health literacy, engagement with healthcare services, and acceptance further influence whether an available intervention is ultimately received [5,7,8,16,35,42]. These factors appear particularly important in explaining disparities within healthcare systems, even in settings where nirsevimab is broadly available.

These levels are interdependent. For example, caregiver-related and socioeconomic barriers may have a smaller effect when prophylaxis is universally funded and routinely offered before neonatal discharge, whereas the same barriers may become more influential when administration requires prescription, pharmacy dispensing, an additional outpatient appointment, transportation, or insurance authorization [5,7,8,16,29,30,31,32,33,34,40,41,42]. Similarly, socioeconomic inequalities may be amplified by fragmented financing and delivery pathways and potentially attenuated by public financing, automatic identification of eligible infants, and proactive recall strategies [5,7,8,16,22,40,42]. Thus, individual-level disparities should not be interpreted independently of the broader policy and organizational environment in which they arise.

The available evidence further suggests that the different determinant categories are not equally supported by the current literature. National policy design and organization of delivery pathways are the determinants most consistently associated with major differences in population-level coverage across implementation settings [5,6,9,10,11,12,13,14,15,16,17,18,19,20,21,22,23,24,25,26,27,28,29,30,31,32,33,34]. In particular, universal eligibility, public financing, reliable procurement, integration into routine neonatal care, and organized catch-up mechanisms have repeatedly accompanied high and relatively homogeneous uptake [19,20,21,22,23,24,25,26,29,30,31,32,33,34]. Financing and reimbursement arrangements also appear important, particularly in systems in which provider stocking, insurance authorization, or reimbursement procedures influence access [5,8,9,36,37,38,39,40,41]. By contrast, demographic, socioeconomic, caregiver, and provider characteristics are important determinants of within-system inequalities, but their magnitude appears more context-dependent and is currently supported by a smaller number of observational studies [5,7,8,16,35,42].

This relative ordering should be interpreted qualitatively rather than as a formal ranking of effect sizes. The available studies differ substantially in populations, healthcare systems, implementation strategies, definitions of uptake, and analytical methods, preventing direct quantitative comparison of the independent contribution of each determinant. Rather, the evidence suggests a pathway extending from policy and financing, through organization and delivery, to individual access and acceptance, with surveillance and registry systems operating across these levels by identifying missed populations and enabling corrective action [5,7,8,16,22,29,30,31,32,33,34,40,41,42]. Successful implementation therefore depends on alignment across multiple levels; weaknesses at any stage may reduce timely and equitable coverage even when the preventive product itself is available and clinically effective.

### 4.6. Provider- and Community-Level Implementation

At the frontline level, successful implementation depends not only on product availability but also on whether healthcare professionals consistently identify eligible infants, recommend prophylaxis, communicate its purpose effectively, and have practical mechanisms for administration and documentation. Variation in provider familiarity with recommendations, uncertainty regarding eligibility, concerns about reimbursement or product availability, and unclear responsibility between maternity, neonatal, and primary-care teams can contribute to missed opportunities even when national policy is favorable [5,7,8,41,42].

Provider recommendation is particularly important because caregiver acceptance of a newly introduced preventive intervention may depend on the clarity, confidence, and consistency with which it is presented. Training, standardized eligibility algorithms, clinical decision support, clear assignment of responsibility, and integration of counseling into routine antenatal, maternity, neonatal, and pediatric encounters may therefore reduce variability in practice. These strategies are especially relevant in dual maternal vaccination–nirsevimab models, in which providers must consider maternal vaccination status, timing of vaccination, infant age, clinical risk, and product availability before determining the appropriate preventive pathway.

Community-level implementation may become increasingly important in settings where hospital delivery does not capture the entire target population or where access to routine pediatric care is fragmented. Community health workers, outreach personnel, and other community-based healthcare providers could potentially support identification of eligible infants, caregiver education, appointment navigation, recall, and linkage with facilities able to administer monoclonal antibody prophylaxis. Such approaches may be particularly relevant in underserved or geographically remote populations. However, direct evidence for community health worker involvement in nirsevimab delivery remains limited, and their role should therefore be evaluated prospectively within locally adapted implementation models rather than assumed to be universally applicable.

### 4.7. From Describing Disparities to Addressing Structural Inequities

Observed differences in nirsevimab uptake according to race and ethnicity, insurance status, income, geographic deprivation, and neighborhood opportunity should not be interpreted as isolated individual-level predictors. Rather, these characteristics may identify populations differentially exposed to structural barriers, including unequal access to primary and neonatal care, fragmented insurance and reimbursement systems, geographic distance from administering facilities, differences in continuity of care, institutional administrative requirements, limited access to trusted information, and unequal opportunities to benefit from preventive services [5,7,8,16,40,42].

Race and ethnicity require particularly cautious interpretation. Lower uptake observed in some racial or ethnic minority populations may reflect structural inequities within healthcare systems rather than biological or cultural characteristics of those populations. Potential mechanisms include differences in access to healthcare facilities, insurance coverage, continuity with trusted providers, language-concordant communication, transportation, institutional trust, and the cumulative effects of discriminatory or exclusionary healthcare experiences. The current evidence does not allow the independent contribution of systemic racism to nirsevimab uptake to be quantified; however, racial and ethnic disparities should prompt investigation of potentially modifiable structural and institutional mechanisms rather than attribution to recipient characteristics alone.

Geographic disparities may similarly arise through differences in maternity-service availability, primary pediatric care density, travel distance, product stocking, transportation, and the capacity of local health systems to identify and recall eligible infants. These barriers may be particularly relevant in rural, remote, and resource-constrained settings, where reliance on facility-based administration alone may leave some infants unreached.

An equity-oriented implementation strategy should therefore combine universal eligibility and access with additional support proportional to the likelihood of being missed. Such a proportionate-universalism approach may include more intensive outreach in deprived or geographically isolated areas, proactive registry-based invitations, removal of out-of-pocket costs, transportation or mobile-service support where appropriate, culturally and linguistically tailored communication, engagement with trusted community organizations, and enhanced follow-up for families with limited access to routine healthcare. Equity indicators should be defined prospectively and monitored alongside overall coverage so that widening disparities can be detected during implementation rather than only after a campaign has concluded.

Community engagement should also extend beyond one-way education. Involving caregivers, community representatives, frontline providers, and local organizations in the design of communication and delivery strategies may help identify practical barriers, improve trust, and ensure that implementation pathways reflect local needs. In settings with established community health worker programs, these personnel may potentially support education, identification of eligible infants, navigation, and linkage to administering facilities, although nirsevimab-specific evidence for this strategy remains limited and requires evaluation.

## 5. WAidid Consensus Recommendations

Based on the available evidence and the collective interpretation of the WAidid expert panel, successful and equitable implementation of long-acting monoclonal antibodies for infant RSV prevention requires coordinated action across policy, financing, healthcare delivery, surveillance, and communication. The following priorities represent the principal consensus recommendations arising from this review.

First, national RSV prevention policies should provide clear and operationally feasible recommendations defining the populations eligible for long-acting monoclonal antibody prophylaxis, the timing and setting of administration, and the responsibilities of maternity, neonatal, and primary pediatric services. Ambiguity regarding eligibility or provider responsibility may contribute to missed opportunities and inconsistent implementation.

Second, sustainable financing and predictable reimbursement mechanisms are essential. Publicly funded or vaccine-like reimbursement models can reduce financial barriers for families and providers, facilitate product stocking, and support more equitable implementation. Procurement strategies should also incorporate long-term planning for product availability and supply chain resilience.

Third, administration should be integrated as closely as possible into routine neonatal and pediatric care pathways. For infants born during the RSV season, administration before discharge from the birth hospital represents an important opportunity to maximize timely protection and reduce loss to follow-up. This strategy should be complemented by structured outpatient pathways for infants born outside the season or those not immunized before discharge.

Fourth, effective programs require linked population registries, recall systems, and real-time surveillance. Integration of birth, maternal immunization, infant immunization, and hospitalization data would facilitate identification of eligible infants, reduce duplication or missed prophylaxis, allow timely assessment of coverage, and enable recognition of geographic and socioeconomic disparities.

Fifth, implementation strategies should incorporate equity as a prospective program objective rather than solely as a retrospective outcome. Universal access should be accompanied by additional support proportional to the risk of being missed. Coverage should therefore be monitored according to socioeconomic, demographic, geographic, and healthcare-access indicators. Where disparities are identified or anticipated, programs should consider intensified outreach, culturally and linguistically appropriate communication, community engagement, removal of financial barriers, proactive registry-based recall, and locally appropriate strategies to overcome geographic and institutional barriers. Differences by race or ethnicity should trigger assessment of structural and healthcare-system mechanisms rather than attribution to individual characteristics.

Sixth, national strategies should consider maternal RSV vaccination and infant monoclonal antibody prophylaxis within the broader context of the local healthcare system rather than assuming that a single strategy will be optimal in all settings. Maternal vaccination has important potential advantages, including protection from birth through transferred maternal antibodies and integration into established antenatal care pathways. Infant monoclonal antibodies, in contrast, provide direct protection independent of maternal vaccine uptake and can be incorporated into neonatal and pediatric services. The relative advantages of these approaches depend on local epidemiology, healthcare infrastructure, maternal vaccination coverage, neonatal care organization, financing, product availability, and population preferences. In some healthcare systems, the two approaches may therefore be complementary rather than simply competing strategies.

Finally, implementation policies should remain adaptable as additional preventive products and new real-world evidence become available. Periodic reassessment of eligibility criteria, procurement strategies, delivery pathways, and cost-effectiveness will be necessary as the RSV prevention landscape evolves.

## 6. Limitations

Several limitations should be considered when interpreting this consensus document. Most importantly, the currently available implementation evidence is derived predominantly from high-income countries, where financing mechanisms, healthcare infrastructure, neonatal delivery systems, immunization registries, and access to primary pediatric care may differ substantially from those in LMICs. Consequently, the magnitude and relative importance of the determinants identified in this review cannot automatically be extrapolated to all settings.

The evidence base is also heterogeneous with respect to study design, population characteristics, definitions of uptake and coverage, healthcare settings, implementation models, follow-up periods, and reported outcomes. This heterogeneity precluded quantitative pooling and limits direct comparisons across countries and programs.

In addition, the evidence reviewed relates predominantly to nirsevimab, because it is currently the long-acting monoclonal antibody for which substantial post-licensure implementation experience is available. Clesrovimab and other emerging products could modify the future implementation landscape, but sufficient real-world evidence concerning their coverage, delivery, financing, and equity implications was not yet available for inclusion in the main analysis.

Maternal RSV vaccination was considered principally as an important contextual determinant of infant monoclonal antibody uptake rather than as a co-primary intervention. Accordingly, this document should not be interpreted as a comparative assessment of the clinical effectiveness, cost-effectiveness, or overall public health value of maternal vaccination versus infant monoclonal antibody prophylaxis.

Finally, evidence on some important implementation domains—including caregiver preferences, provider behavior, equity interventions, and health-system performance—is still limited or derived from individual healthcare systems. The associations identified should therefore be interpreted in light of the local policy and healthcare context.

## 7. Research Priorities and Future Directions

The rapid evolution of RSV prevention requires a prospective research agenda extending beyond measures of efficacy and effectiveness. Over the next 3–5 years, implementation research should address the following major evidence gaps.

### 7.1. Implementation in Low- and Middle-Income Countries

Dedicated implementation studies in LMICs should be a major priority. These settings account for a substantial proportion of the global burden of severe infant RSV disease but remain underrepresented in the current evidence base.

Future studies should determine how the implementation of long-acting monoclonal antibodies is influenced by product pricing and affordability, procurement capacity, storage and distribution requirements, healthcare workforce constraints, access to antenatal and neonatal services, fragmented referral pathways, limited population registries, and surveillance capacity. Rather than assuming that models developed in high-income settings can be transferred directly, studies should identify locally feasible delivery pathways and financing mechanisms.

Where direct experience with RSV monoclonal antibodies remains limited, implementation research may also consider lessons from other maternal immunization programs, birth-dose preventive interventions, and biologic products delivered through maternal, neonatal, and pediatric healthcare systems.

### 7.2. Real-World Economic Evaluation and Sustainable Financing

Economic evaluations should increasingly incorporate real-world implementation costs rather than acquisition price alone. Relevant costs include procurement, distribution, storage, staff time, inventory management, reimbursement administration, surveillance infrastructure, recall systems, and the resources required to reach populations with limited healthcare access.

Cost-effectiveness analyses should also compare alternative delivery strategies, including universal and risk-based programs, hospital-based and outpatient delivery, maternal vaccination, infant monoclonal antibody prophylaxis, and mixed strategies. Particular attention should be given to how different financing and reimbursement models influence both aggregate health outcomes and equity.

### 7.3. Evaluation of Maternal Vaccination–Nirsevimab Strategies

Further research is needed to determine how maternal RSV vaccination and infant monoclonal antibody prophylaxis can be optimally combined within different healthcare systems.

Maternal vaccination offers the operational advantage of integration into antenatal care and the potential for infant protection from birth through transplacental antibody transfer. Infant monoclonal antibody prophylaxis provides an alternative pathway for infants whose mothers were not vaccinated or when maternal immunization did not occur sufficiently before delivery, and may be particularly suitable for healthcare systems with strong neonatal delivery infrastructure.

Future studies should therefore evaluate mixed maternal vaccine–nirsevimab models, including their effects on overall population protection, timeliness, missed opportunities, caregiver preferences, healthcare utilization, program complexity, cost-effectiveness, and equity. Such studies should identify which combinations are most appropriate under different epidemiological, organizational, and economic conditions rather than assuming universal superiority of either strategy.

### 7.4. Equity of Access

Future implementation studies should move beyond reporting overall coverage and systematically examine who remains unprotected and why. Analyses should assess differences according to socioeconomic status, insurance coverage, race and ethnicity where appropriate, geographic deprivation, migration status, healthcare access, and other locally relevant determinants.

Interventional studies are particularly needed to evaluate whether measures such as hospital-based administration, proactive registry-based recall, community outreach, culturally adapted communication, reduced out-of-pocket costs, and simplified reimbursement can measurably reduce disparities.

Equity indicators should ideally be incorporated prospectively into program monitoring systems rather than assessed only after disparities have emerged.

### 7.5. Data Linkage and Implementation Surveillance

A major structural research and policy priority is the development of interoperable systems linking maternal vaccination records, birth registries, infant immunization records, primary-care data, and RSV-related healthcare outcomes.

Such linkage is particularly important in healthcare systems using both maternal vaccination and infant monoclonal antibodies, where determination of infant eligibility may depend on maternal vaccination status and timing. Future studies should assess whether integrated digital systems reduce duplication, missed opportunities, delayed administration, and inequalities in coverage.

Standardized definitions of eligibility, uptake, timely administration, complete protection, missed opportunities, and equity outcomes would also facilitate comparisons across countries and programs.

### 7.6. Emerging Long-Acting Monoclonal Antibodies

The introduction of additional long-acting monoclonal antibodies, including clesrovimab, will require renewed evaluation of implementation strategies. Real-world studies should assess uptake, effectiveness, safety, delivery pathways, provider and caregiver acceptance, reimbursement, and equity as these products enter routine practice.

The availability of more than one long-acting monoclonal antibody could also potentially influence procurement and economic conditions. Increased product availability and competition may create opportunities for alternative procurement strategies, improved price sustainability, diversification of supply, and greater supply chain resilience. These potential benefits, however, remain to be demonstrated empirically and should be evaluated as real-world implementation data accumulate.

Comparative implementation studies should therefore examine not only clinical outcomes but also acquisition costs, contracting and procurement models, reliability of supply, ease of administration, programmatic costs, and population reach.

## 8. Conclusions

The public health impact of long-acting monoclonal antibodies for infant RSV prevention depends not only on their biological efficacy but on whether healthcare systems can deliver them timely, consistently, sustainably, and equitably. Current experience with nirsevimab demonstrates that high coverage is most likely when clear national recommendations are supported by sustainable financing, reliable procurement, integration into routine neonatal and pediatric care, structured catch-up pathways, population registries, and active surveillance of implementation and equity.

No single RSV prevention strategy is likely to be optimal in every healthcare system. Maternal vaccination and infant monoclonal antibody prophylaxis have different operational strengths and constraints, and their relative roles should be determined according to local epidemiology, healthcare organization, financing, access to antenatal and neonatal care, and population preferences. The purpose of this consensus document is therefore not to establish the superiority of monoclonal antibodies over maternal vaccination, but to define the conditions required to achieve successful and equitable implementation when long-acting monoclonal antibodies are incorporated into infant RSV prevention programs.

The major challenge for the next phase of RSV prevention is to translate highly effective preventive products into equitable population-level protection. This will require closing evidence gaps in LMICs, developing sustainable financing and procurement models, integrating maternal and infant immunization data, evaluating mixed maternal vaccination–monoclonal antibody strategies, and ensuring that emerging products expand rather than exacerbate inequalities in access. These priorities should guide implementation research and policy development over the coming years as the range of available RSV preventive options continues to expand.

## Figures and Tables

**Table 1:** National policy models and their impact on nirsevimab uptake.

Policy Model	Main Characteristics	Impact on Nirsevimab Uptake	Key Implications
Maternal RSV vaccination prioritized	RSV prevention strategy mainly based on vaccination during pregnancy; nirsevimab reserved for selected high-risk infants or specific clinical situations.	Limited or negligible population-level nirsevimab uptake during early implementation seasons.	Reduces the target population for nirsevimab and may simplify antenatal prevention, but infant protection depends on maternal vaccine uptake and equity.
Dual-strategy model	Either maternal RSV vaccination or infant nirsevimab recommended, with most infants expected to receive only one intervention.	Moderate and heterogeneous uptake, influenced by maternal vaccine coverage, supply, reimbursement, and delivery setting.	Requires clear guidance, coordination between obstetric and pediatric services, and monitoring to avoid missed protection.
Universal infant monoclonal antibody program	Nirsevimab offered to all eligible infants, usually seasonally and publicly funded.	Highest reported coverage, often approaching or exceeding 90% when integrated into neonatal care.	Supports rapid and equitable protection when financing, logistics, and delivery pathways are well organized.
Risk-based infant program	Nirsevimab restricted to infants at increased risk of severe RSV disease.	Lower population-level uptake by design.	May reduce costs but limits broader public health impact and may miss infants without recognized risk factors.
Regionally implemented or transitional programs	Programs introduced at regional level or during early rollout phases before national scale-up.	Initial moderate uptake followed by higher coverage when programs became more structured.	Demonstrates the importance of operational maturity, registry use, and integration into routine care.

**Table 2:** Organizational determinants of nirsevimab implementation.

Organizational Factor	How This Influences Uptake	Expected Effect on Coverage
Administration before hospital discharge	Captures infants immediately after birth and avoids reliance on later outpatient visits.	Increases timely uptake and minimizes missed opportunities.
Integration into routine neonatal care	Embeds nirsevimab within established maternity and newborn workflows.	Enables high and homogeneous coverage.
Population registries and recall systems	Identify infants born outside the RSV season and invite them for scheduled immunization.	Improves protection of catch-up cohorts.
Coordination between maternity and primary pediatric care	Ensures continuity between birth hospitalization and outpatient follow-up.	Reduces gaps among infants not immunized at birth.
Outpatient-only or fragmented delivery	Requires prescription, appointment scheduling, caregiver adherence, dispensing, and administration across multiple providers.	May reduce timeliness and increase variability.
Clear provider responsibility	Defines who should offer, prescribe, administer, and record nirsevimab.	Improves accountability and program consistency.
Reliable product supply and distribution	Prevents delays, shortages, or uneven availability across sites.	Supports consistent access across regions and facilities.
Surveillance and coverage monitoring	Allows identification of low-coverage groups and geographic gaps.	Enables corrective interventions and equity-focused planning.

**Table 3:** Determinants of suboptimal or unequal nirsevimab uptake.

Determinant	Examples	How It Affects Uptake	Possible Strategies to Increase Uptake
Policy framework	Maternal vaccination prioritized; risk-based eligibility; lack of universal recommendation.	Narrows eligibility or creates uncertainty among providers and caregivers.	Clear national recommendations; harmonized guidance across obstetric and pediatric services.
Financing and reimbursement	High acquisition cost; insurance-dependent reimbursement; delayed payment to providers.	Limits stocking, administration, and equitable access.	Public funding; vaccine-like reimbursement pathways; reduced provider financial risk.
Delivery pathway	Outpatient-only administration; pharmacy-based dispensing; multi-step access.	Increases missed opportunities and delays.	Hospital-based administration at birth; integrated catch-up programs.
Healthcare access	Limited primary care availability; geographic barriers; fragmented services.	Reduces likelihood of timely administration.	Outreach programs; mobile clinics; registry-based invitations.
Socioeconomic status	Lower income; public insurance; neighborhood deprivation.	Associated with lower uptake despite product availability.	Targeted communication; no-cost access; proactive recall of underserved populations.
Caregiver health literacy	Limited awareness of RSV severity or nirsevimab benefits.	Reduces acceptance and intention to immunize.	Culturally appropriate education; provider-led counseling.
Race and ethnicity	Lower uptake reported among some minority groups in real-world studies.	Reflects structural inequities and unequal healthcare access.	Equity monitoring; community engagement; tailored interventions.
Timing relative to RSV season	Infants born before the season may not be reached at birth.	Requires catch-up administration through outpatient services.	Seasonal registries; early appointments; systematic recall.
Provider awareness and practice	Variable familiarity with recommendations or uncertainty about eligibility.	Leads to inconsistent counseling and administration.	Training, clinical decision support, standardized protocols.
Surveillance infrastructure	Limited real-time coverage data.	Delays recognition of implementation gaps.	Linked birth, immunization, and hospitalization databases.

## Data Availability

No new data were created or analyzed in this study.
